# Diverse reactions of aquaculture biofilter biofilms following acute high-dose peracetic acid

**DOI:** 10.1016/j.bioflm.2025.100277

**Published:** 2025-04-02

**Authors:** Wanhe Qi, Sanni L. Aalto, Peter Vilhelm Skov, Kim João de Jesus Gregersen, Lars-Flemming Pedersen

**Affiliations:** a Technical University of Denmark, DTU Aqua, Section for Aquaculture, The North Sea Research Centre, P.O. Box 101, DK-9850, Hirtshals, Denmark; b Natural Resources Institute Finland (Luke), Survontie 9A, FI-40500, Jyväskylä, Finland

**Keywords:** Disinfection, Microbial inhibition, Nitrification, Flow cytometry, Respirometry, RAS

## Abstract

Peracetic acid (PAA) is an effective disinfectant in aquaculture systems to reduce pathogen loads and improve water quality. However, its effectiveness in disinfecting biofilm in recirculating aquaculture systems (RAS) and resetting biofilters between productions remains unknown. This study evaluated the effects of acute PAA exposure on biofilter biofilms from freshwater RAS. Identical types of bioelements were collected from a pilot-scale RAS (without prior PAA treatment) and a commercial RAS (with PAA treatment), and exposed to PAA concentrations of 0, 1, 2, 4, 8, and 16 mg/L for 1 h. Microbial activity and viability of the exposed biofilms were evaluated using respirometry and flow cytometry. Results showed dose-dependent inhibition of biofilm activity and viability in the pilot-scale RAS. Nitrite oxidation was the most sensitive process to PAA, with an IC_50_ of 1.27 mg/L (the concentration at which PAA inhibited biofilm metabolic activity by 50 %), followed by ammonia oxidation (IC_50_ = 1.59 mg/L) and endogenous respiration (IC_50_ = 2.67 mg/L). Microbial activity linked to H_2_O_2_ decomposition was least affected (IC_50_ = 4.68 mg/L). Live cell counts decreased from 9.1 × 10^7^ counts/cm^2^ to 2.4 × 10^7^ counts/cm^2^ of bioelement surface, with dead cells proportion increasing from 15 % to 54 %. In contrast, biofilter biofilms from the commercial RAS exhibited significantly lower sensitivity to PAA dosage, with reductions in nitrite oxidation (39 %) and ammonia oxidation (51 %) observed only at 16 mg/L compared to control. These findings suggest that routine PAA exposure, as part of the other operating conditions on the commercial RAS, can enhance the biofilm's sensitivity to PAA. The study provides new insight into the sensitivity of aquaculture biofilm to PAA treatment and its effect on associated microbial processes.

## Highlights

• Biofilm activity and viability were quantified by respirometry and flow cytometry.
• Nitrite oxidation was the most sensitive microbial process to PAA exposure.
• Significant PAA's dose-dependent inhibitory effect on biofilm from pilot-scale RAS.
• Biofilm from a commercial RAS with regular PAA use were less sensitive to PAA.

## 1 Introduction

Biofilm, an aggregate of microorganisms and their self-produced matrix attached to a surface [1], can play both beneficial and detrimental roles in natural and engineered systems, depending on the location and the species involved [2,3]. This also applies to recirculating aquaculture systems (RAS), an environmental controlled land-based facility for intensive fish farming with a high-degree water reuse during the production [4]. RAS biofilms include prokaryotes and eukaryotes, autotrophs and heterotrophs, as well as aerobes and anaerobes, depending on the categorization [[5], [6], [7], [8], [9]]. For example, biofilm growing on biofilter elements hosts autotrophic ammonia-oxidizing bacteria (AOB) and nitrite-oxidizing bacteria (NOB), performing nitrification process to maintain proper water quality for fish growth [6]. However, biofilm can also act as a refuge for pathogenic bacteria, sheltering them from antibiotics or disinfectants treatment, which enables them to survive and subsequently recolonize the water phase, posing a significant risk during the production [[10], [11], [12], [13]]. Therefore, RAS operation also includes biofilm management to ensure a healthy and stable production environment for fish.

One strategy to control the microbial water quality and prevent undesirable biofilm formation in RAS is through the application of chemical disinfectants [[14], [15], [16], [17]]. Disinfection is a central component in RAS hygiene procedure to prevent pathogen introduction, accumulation, and transmission within and between facilities [18,19]. Several chemical disinfectants have been used for water disinfection in RAS, and peracetic acid (PAA) is a commonly used disinfectant [20,21], due to its rapid degradation into harmless residues and broad-spectrum antimicrobial effects on cyst-forming dinoflagellates, parasites, planktonic bacteria, and sessile biofilm [15,[22], [23], [24], [25], [26], [27]].

Previous studies have reported that PAA can harm nitrifying biofilm in RAS when applied at a dose of 2–3 mg/L PAA or at repeated doses of 1.1 mg/L PAA twice daily, four times a week [28,29]. The potential inhibitory effect of PAA on nitrifying biofilm is a critical factor in the utilization of PAA as a disinfectant in RAS. As RAS rely on a high degree of water reuse and constant water circulation, any residual PAA making contact with the biofilter units may inactivate the beneficial biofilm within the biofilter, resulting in impaired biofilter performance and deteriorated system water quality.

PAA is also used in RAS to disinfect biofilters between production batches. Particularly because the biofilter may serve as a reservoir for pathogens, it can be relevant to chemically disinfect the biofilter at the end of a production cycle when no fish are present [30]. After disinfection and resetting the biofilter, a new production cycle can be initiated. In this regard, the operators are concerned about the efficiency of the disinfectants on the biofilters, specifically how many microorganisms are inactivated or survive after disinfection. Information about reductions of microbial presence and processes in biofilm (i.e., viability and metabolic activities) in response to PAA exposure is required to optimize and standardize PAA dosage but is currently missing due to the complexity of biofilm and the lack of suitable methods to characterize biofilm.

Existing methods to quantify biofilm activity and its viability in RAS include controlled spiking batch test (i.e., measure the turnover rates of ammonium or nitrite to nitrate; [31,32]), plate counting (i.e., measure colony forming units in the culture; [33]), next-generation sequencing (i.e., measure microbial community composition and function; [34,35]), and qPCR (quantitative polymerase chain reaction, i.e., measure gene expression; [29,36]). These methods are either time- and labor-intensive or they require specific expertise. Recently developed respirometric methods based on measuring oxygen changes allow for a simple and fast assessment of biofilm metabolic activities inside the biofilter without destroying biofilm integrity [37,38]. Additionally, the feasibility of applying flow cytometry for fast and precise determination of microbial viability in microbes suspended in RAS water was demonstrated by Aalto et al. [39], a method that can also be applied to assess microbial viability in biofilm samples. Both respirometry and flow cytometry methods appear to be suitable options to characterize biofilm on biofilter elements and obtain the information on the effects of PAA on biofilm inside RAS biofilters.

The objective of this study was therefore to evaluate the effects of PAA on biofilm from biofilter elements sampled at two different RAS facilities (a pilot-scale and a commercial RAS), applying new methods to quantify the metabolic activities and viability of biofilm.

## 2 Materials and methods

### 2.1 Bioelement samples

Colonized bioelements were collected from moving bed biofilters at two different freshwater RAS facilities with rainbow trout: 1) a pilot-scale RAS at DTU Aqua (Hirtshals, Denmark) and 2) an outdoor commercial RAS (Farsø, Denmark). Both biofilters were filled with black RK Plast bioelements made of polypropylene with a specific surface area of 750 m^2^/m^3^ (Dania Plast A/S, Skive, Denmark). The biofilter and 20 m^3^ pilot-scale RAS are described in detail in Aalto et al. [40]. The commercial freshwater RAS had an annual production capacity of 600 MT rainbow trout with a 450 m^3^ moving bed biofilter.

The disinfection protocol at the commercial RAS included regular PAA application (every second day for years), whereas no chemical disinfection had been applied in the pilot-scale RAS. Unlike the experimentally controlled and stable indoor pilot-scale RAS, the commercial RAS was subjected to seasonal changes and operational variability (e.g., light exposure, temperature, DO, and pH).

### 2.2 Acute PAA exposure experiment

The acute exposure experiment was conducted in six 50 L containers containing tap water, where 25 pieces of collected bioelements were placed and exposed to the targeted PAA concentrations for 1 h. The tested PAA concentrations in the experiment were 0, 1, 2, 4, 8, and 16 mg PAA/L, and achieved by the addition of PAA into the corresponding container, using an industrial PAA solution (Aqua-Oxides Super, S. Sørensen, Thisted, Denmark). The composition of the PAA product was 15.7 % of PAA and 25.8 % of hydrogen peroxide (H_2_O_2_) as weight percentage, which was measured by a two-step titration method [28].

After 1-h exposure, the 25 pieces of bioelements exposed to each specific PAA concentration were collected separately and instantly transferred to separate static beakers containing 1 L of fresh RAS water for 30 min to eliminate residual PAA. The RAS water used for neutralization was collected from the facilities where the bioelements were sampled. To account for potential cell detachment from biofilm during the neutralization, concurrent controls were included. These controls consisted of bioelements exposed to the same handling procedures but without PAA treatment (0 mg PAA/L). This allowed differentiation between cells lost due to detachment during neutralization and cells impacted by PAA exposure. After the neutralization step, the exposed bioelements were collected to measure biofilm metabolic activity and viability with respirometry and flow cytometry methods, respectively. All experiments were performed in triplicate, with new bioelements collected for each replicate to ensure biological replication. The experiment was operated in a temperature-controlled room, and experimental temperature was 16 ± 1 °C.

### 2.3 Biofilm metabolic activity measurement

The metabolic activities of biofilm on bioelements were measured using respirometric methods described in Qi et al. [37,38]. Briefly, biofilm activities related to endogenous respiration, nitrite oxidation, and ammonia oxidation processes were assessed as a proxy of oxygen consumption rates (OCR) using pure tap water, or sequential spiking with NaNO_2_ or NH_4_Cl. The microbial activity in the biofilm was based on enzymatic H_2_O_2_ decomposition [41], which was indirectly evaluated as the net oxygen release rate (*k*_*or*_) following a spike with H_2_O_2_ [38]. Fiber optic oxygen sensors (FireSting, PyroScience GmbH, Germany) were used to record real-time dissolved oxygen (DO) concentrations. The nominal concentration of NH_4_Cl, NaNO_2_ and H_2_O_2_ injected into chambers were 11.5 mg/L (∼3 mg NH_4_^+^-N/L), 14.8 mg/L (∼3 mg NO_2_^−^-N/L) and 10 mg/L, respectively. The detailed procedures are described in Qi et al. [42].

### 2.4 Flow cytometry analysis of biofilm viability

One exposed bioelement was placed in a sterilized Petri dish and the biofilm was detached by scraping the eight lamella surfaces of the bioelement with a sterile sickle scale. After that, 5 mL sterilized buffered sodium chloride peptone broth of 16.1 g/L was used to thoroughly rinse the scraped surfaces of bioelements five times to get the remaining biofilm detached by using a syringe with pointed end tip needle. After dislodging the biofilm, the obtained cell suspension was collected by aspiration with a pipette, and then filtered through a 40-μm sterile cell strainer (Fisherbrand™, Fisher Scientific) to remove large particles that can interfere with flow cytometry analysis. After filtration, the 500 μL filtrate was then labeled with two nucleic acid stains simultaneously, cell membrane-permeant SYBR Green and cell membrane impermeant propidium iodide (PI) to distinguish live and dead cells and measured with BD Accuri C6 Plus flow cytometer (Becton, Dickinson and Company, NJ, USA), following the protocols from Aalto et al. [34].

### 2.5 Data and statistical analysis

#### 2.5.1 Oxygen consumption rate (OCR) calculation following dissolved nitrogen substrate injection

The OCR was calculated as described previously [37]:

(1) $$\text{OCR}=V\times \frac{\Delta \text{DO}}{\Delta t}$$

where OCR (mg O_2_/h) is the oxygen consumption rates of biofilm that utilizes the substrate. V (=0.35 L) is the volume of the chamber for DO measurement, *t* (h) is the period for calculating OCR, and $\frac{\Delta \text{DO}}{\Delta t}$ is the change in DO concentration over time deduced from a linear regression model.

Specifically, the OCR of biofilm in the absence of substrate, and in the presence of NH_4_Cl and NaNO_2_ were calculated in the following:

(2) $${\text{OCR}}_{\text{endo}}={\text{OCR}}_{\text{tap}\;\text{water}\;\text{spike}}$$

(3) $${\text{OCR}}_{\text{NOB}}={\text{OCR}}_{\text{Na}{\text{NO}}_{2}\;\text{spike}}‐{\text{OCR}}_{\text{endo}}$$

(4) $${\text{OCR}}_{\text{AOB}}={\text{OCR}}_{{\text{NH}}_{4}\text{Cl}\;\text{spike}}‐{\text{OCR}}_{\text{endo}}$$

where OCR_endo_ is the oxygen consumption rate of biofilm in pure tap water, reflecting the endogenous respiration activity of biofilm. OCR_NOB_ is the oxygen consumption rate of NOB in the biofilm, it reflects the biofilm metabolic activity for nitrite oxidation. OCR_AOB_ is the oxygen consumption rate of AOB in the biofilm, it reflects the biofilm metabolic activity for ammonia oxidation.

OCR_tap water spike_, ${\text{OCR}}_{\text{Na}{\text{NO}}_{2}\;\text{spike}}$ and ${\text{OCR}}_{{\text{NH}}_{4}\text{Cl}\;\text{spike}}$ are the oxygen consumption rates of biofilm related to tap water, NaNO_2_, and NH_4_Cl spike, respectively.

#### 2.5.2 Oxygen release rate (k_or_) calculation following H_2_O_2_ injection

The k_or_ was calculated with a monomolecular model based on the work of Qi et al. [38]:

(5) $$y=A\left(1‐e^{{‐k}_{or}\left(t‐t_{c}\right)}\right)$$

Where y (mg O_2_/L) is the DO concentration in the chambers following H_2_O_2_ spike measured by oxygen sensors, A (mg O_2_/L) was the maximum net oxygen release potential, *k*_*or*_ (h^−1^) was the net oxygen release rate constant, *t* (h) was the time of calculation period, and *t*_*c*_ (h) represented the duration of lag phase.

#### 2.5.3 IC_50_ calculation

To investigate which metabolic activity of biofilm is more sensitive to PAA treatment, the median inhibition concentration (IC_50_), the concentration of PAA resulted in a 50 % decrease in metabolic activity of biofilm, is estimated based on a four-parameter logistic model [43] (Eq. (6))

(6) $$y=\frac{A-D}{1+{\left(\frac{x}{C}\right)}^{B}}+D$$

Where y is the relative response (%) of biofilm metabolic activities as compared to the unexposed control at x concentration of PAA (mg/L), while A (%) and D (%) are the responses at the maximum curve asymptote and minimal curve asymptote, respectively. C is the IC_50_ and B is a slope factor. Here, A and D were constrained to be 100 % and 0 %.

#### 2.5.4 Statistical analysis

The data processing, plotting, and fitting of curves were done by using OriginPro software (version 2021, OriginLab, Northampton, USA). A one-way analysis of variance (ANOVA) in OriginPro software following *post-hoc* Tukey test was conducted to test the mean differences of biofilm metabolic activity and biofilm viability among PAA levels. Before ANOVA analysis, the assumptions of normality of residuals and homogeneity of variance were checked by Shapiro-Wilk and Levene's test, respectively. In cases where assumptions were not met, data were analyzed by using the nonparametric Kruskal-Wallis ANOVA, followed by Dunn's multiple comparison test. Statistical significance was set at *P* < 0.05.

## 3 Results

### 3.1 Biofilm activity following acute PAA exposure

**Fig. 1:**
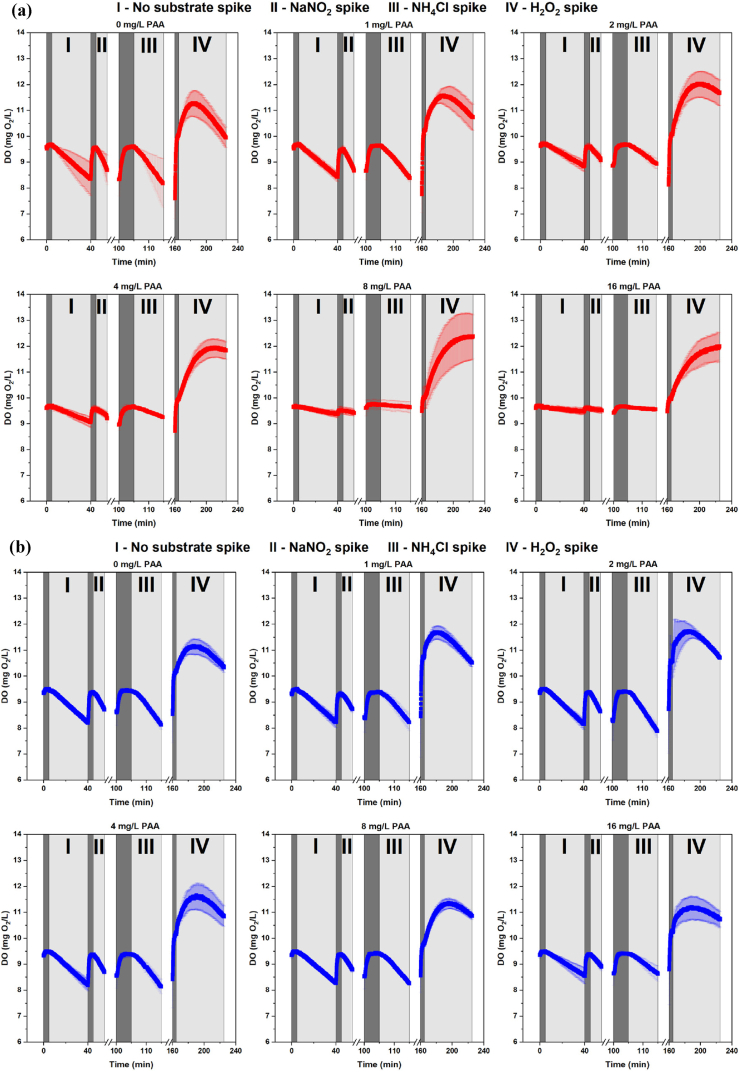
Profiles of dissolved oxygen (DO) concentrations in 350 mL respirometric chambers with 20 pieces of bioelements, collected from **(a)** pilot-scale RAS and **(b)** commercial RAS. First, the bioelements were exposed to different PAA concentrations for 60 min. Then, four sets of substrate spikes were made: first with tap water (I, Time = 0–40 min), then with addition of NaNO_2_ (II, Time = 40–55 min), NH_4_Cl (III, Time = 100–115 min), and H_2_O_2_ (IV, Time = 160–225 min). DO values are represented as mean ± SD (n = 3). Dark shaded areas represent the *pump-on* period when substrate injected into the chambers, light dark shaded areas represent the *pump-off* period when oxygen consumption/release rates were calculated, and white areas with breaks represent intervals of 45 min when the draining and refilling of the reservoir takes place.

In the control group (0 mg PAA/L), an apparent oxygen consumption was observed in the bioelements collected from two locations following the spike with pure tap water and water spiked with NaNO_2_ or NH_4_Cl (Fig. 1). This was observed also when exposing biofilms from pilot-scale RAS to 1, 2, and 4 mg/L of PAA, but not at PAA concentrations of 8 and 16 mg/L (Fig. 1a). In contrast, biofilm samples from the commercial RAS still clearly consumed oxygen after being exposed to PAA even at a concentration of 16 mg/L (Fig. 1b). After H_2_O_2_ spike, the activity of biofilm samples of control group from two locations both resulted in a distinct oxygen release, and the same was observed in biofilms in the bioelements collected from two locations when exposed to PAA (Fig. 1).

**Fig. 2:**
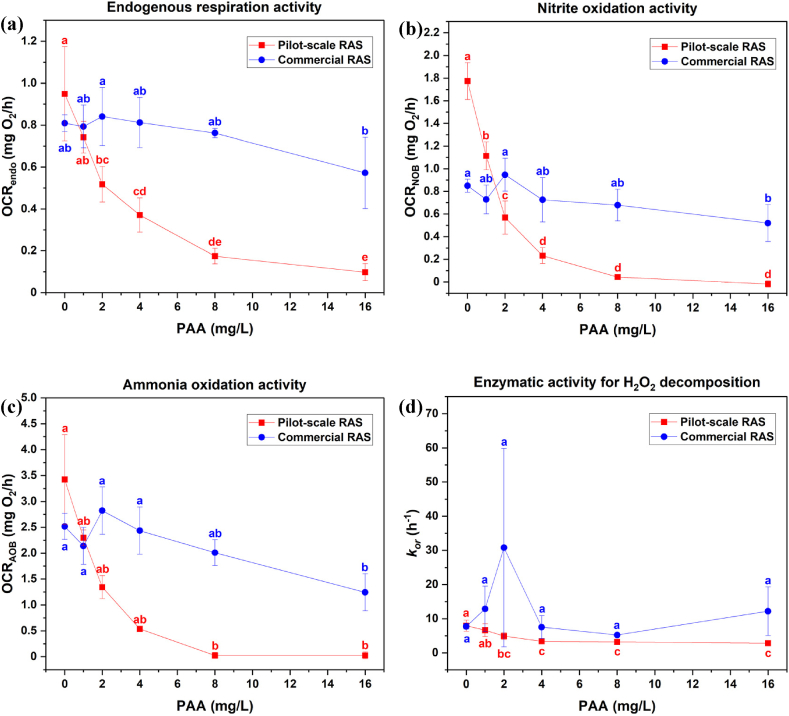
The metabolic activity of biofilm attached on bioelements collected from pilot-scale RAS (red square) and commercial fish farm (blue circle) towards increasing PAA exposure. **(a)** OCR_endo_, **(b)** OCR_NOB_, **(c)** OCR_AOB,_ and **(d)***k*_*or*_ are response parameters indicating biofilm metabolic activities related to endogenous respiration, nitrite oxidation, ammonia oxidation, and enzymatic process for H_2_O_2_ decomposition, respectively. Values are reported as mean ± SD (n = 3). The letters denote *post-hoc* Tukey test results within the PAA concentration level. The same letter represents no significant difference and vice versa (*p* < 0.05).

The endogenous respiration rate (OCR_endo_) of unexposed bioelements after tap water spike, was 0.95 ± 0.22 mg O_2_/h in the pilot-scale RAS and 0.81 ± 0.04 mg O_2_/h in the commercial RAS (Fig. 2a). There was a clear trend that OCR_endo_ of biofilm in bioelements collected from pilot-scale RAS decreased with increasing PAA exposure (r = −0.809, p < 0.0001). A significant decrease by 45 % in OCR_endo_ occurred when bioelements were exposed to 2 mg/L PAA, and the highest reduction of OCR_endo_ in comparison with control was found at 16 mg/L PAA exposure (90 % of OCR_endo_ reduction) (Fig. 2a). The OCR_endo_ of biofilm in bioelements collected from the commercial RAS was only found to decline significantly when being exposed to 16 mg/L PAA, resulting in a 30 % of OCR_endo_ reduction as compared to the control group (Fig. 2a).

In the control group, the oxygen consumption from nitrite oxidation activity (OCR_NOB_) was 1.77 ± 0.16 mg O_2_/h in the pilot-scale RAS and 0.85 ± 0.06 mg O_2_/h in the commercial RAS (Fig. 2b). A significant reduction of OCR_NOB_ was observed when bioelements from pilot-scale RAS were exposed to 1 mg/L PAA, leading to a 37 % decrease in OCR_NOB_ as compared to the control (Fig. 2b). In comparison with control, 16 mg/L PAA exposure lead to almost 100 % reduction of OCR_NOB_ in bioelements collected from pilot-scale RAS. The significant reduction of OCR_NOB_ in bioelements collected from the commercial RAS was only observed at 16 mg/L PAA exposure, leading to a 39 % OCR_NOB_ decline as compared to the control (Fig. 2b).

The ammonia oxidation activity (OCR_AOB_) of the control biofilm was 3.42 ± 0.86 mg O_2_/h in the pilot-scale RAS and 2.52 ± 0.13 mg O_2_/h in the commercial RAS (Fig. 2c). When exposed to increasing PAA concentration, the OCR_AOB_ of biofilter biofilms from pilot-scale RAS decreased by 33 %, 61 %, 84 %, 99 %, and 99 % at 1, 2, 4, 8, and 16 mg/L PAA as compared to the control, respectively (Fig. 2c). The OCR_AOB_ of biofilter biofilms from commercial RAS reduced significantly only when being exposed to 16 mg/L PAA, resulting in a decrease of OCR_AOB_ of 51 % in comparison with control (Fig. 2c).

The microbial activity (*k*_*or*_) of the biofilm in the unexposed bioelements was 7.97 ± 1.64 h^−1^ in the pilot-scale RAS and 7.75 ± 0.80 h^−1^ in the commercial RAS (Fig. 2d). The microbial activity in bioelements from the pilot-scale RAS was significantly reduced (by 38 %) when exposed to 2 mg/L PAA, and the highest reduction of microbial activity by 64 % was observed at 16 mg/L PAA exposure in comparison with control (Fig. 2d). The microbial activity in biofilm from the commercial RAS biofilter varied considerably after PAA exposure (Fig. 2d). Compared to unexposed control biofilm, the microbial activity increased almost three-fold after 2 mg/L PAA exposure, with large variation between the tested bioelements. The mean microbial activity increased by 66 % and 57 % at 1 and 16 mg/L PAA but decreased by 3 % and 33 % at 4 and 8 mg/L PAA respectively, compared to the control.

The inhibition concentration (IC_50_) of PAA for metabolic processes in biofilm samples from pilot-scale RAS followed the order: nitrite oxidation, ammonia oxidation, endogenous respiration, and enzymatic H_2_O_2_ decomposition (Table 1). The dose-response relationship was very high (R^2^ > 0.9) on biofilm samples collected from the pilot-scale RAS, but this relationship was not found on biofilm samples from commercial RAS (Table 1). Within the PAA exposure range tested, 50 % inhibition for the selected metabolic processes on biofilm samples from commercial RAS were not observed (Fig. 2).

**Table 1:** IC_50_ (mg/L) of PAA for different metabolic processes within biofilm samples collected from pilot-scale RAS and commercial RAS.

Metabolic processes	Parameters	Pilot-scale RAS		Commercial RAS	
		IC_50_ (mg/L)^a^	R^2^	IC_50_ (mg/L)	R^2^
Endogenous respiration	OCR_endo_	2.67 ± 0.14	0.99	─	─
Ammonia oxidation	OCR_AOB_	1.59 ± 0.05	0.99	─	─
Nitrite oxidation	OCR_NOB_	1.27 ± 0.02	0.99	─	─
Enzymatic H_2_O_2_ decomposition	*k*_*or*_	4.68 ± 1.06	0.92	─	─

a The data were deduced from fitting dose-response curve with four parameter logistic model, and represented as mean ± SD (n = 3).

### 3.2 Biofilm viability alterations following acute PAA exposure

**Fig. 3:**
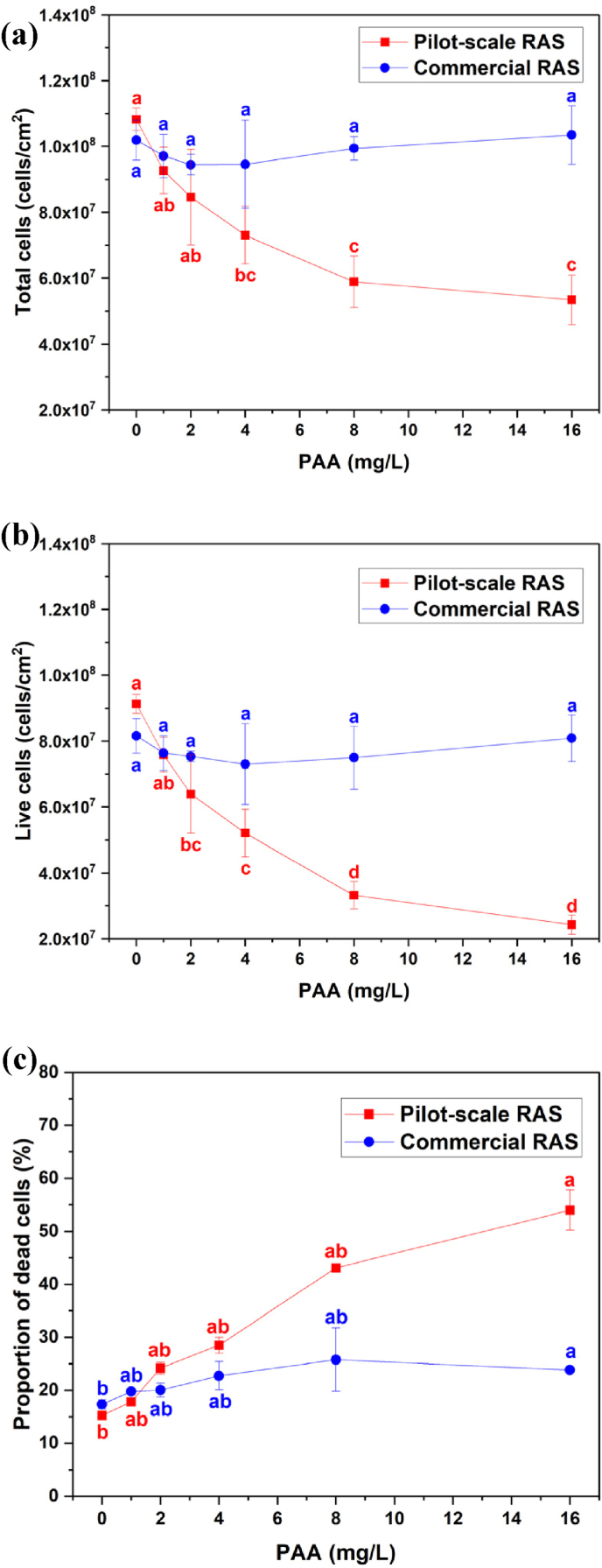
The abundance of **(a)** total cells and **(b)** live cells, and **(c)** the proportion of dead cells (%) in the biofilm that are attached on bioelements collected from pilot-scale RAS (red square) and commercial RAS (blue circle) towards increasing PAA concentration exposure. The biofilm samples for the measurement are dislodged from the surface of a single bioelement covering a surface area of 448 mm^2^.

The abundance of total cells measured in the unexposed bioelements was 1.08 × 10^8^ cells/cm^2^ in the pilot-scale RAS and 1.02 × 10^8^ cells/cm^2^ in the commercial RAS (Fig. 3a). When exposed to PAA, the abundance of total cells in biofilm samples collected from pilot-scale RAS decreased with increasing PAA concentration exposure, in which 4 mg/L PAA exposure started to cause a significant reduction of total cells abundance by 32 %, and the highest reduction of 51 % total cells abundance was observed at 16 mg/L PAA exposure in comparison with control (Fig. 3a). The total cell abundance in biofilm samples collected from commercial fish farm varied between 9.4 × 10^7^ and 1.03 × 10^8^ cells/cm^2^ when exposed to elevated PAA concentrations (Fig. 3a).

The abundance of live cells in biofilm in the unexposed bioelements was 9.1 × 10^7^ counts/cm^2^ in the pilot-scale RAS and 8.2 × 10^7^ cells/cm^2^ in the commercial RAS (Fig. 3b). When exposed PAA, the abundance of live cells in biofilm samples collected from pilot-scale RAS declined with increasing PAA concentrations, ranged from 2.4 × 10^7^ to 7.6 × 10^7^ cells/cm^2^, and 2 mg/L PAA exposure started to cause a significant drop of live cells abundance, resulting in 30 % reduction of live cells abundance compared to control (Fig. 3b). The live cell abundance in biofilm samples collected from commercial RAS varied from 7.3 × 10^7^ to 8.1 × 10^7^ cells/cm^2^ when exposed to elevated PAA concentrations (Fig. 3b).

The proportion of dead cells in the unexposed bioelements was 15.2 ± 0.3 % in the pilot-scale RAS and 17.3 ± 0.9 % in the commercial RAS (Fig. 3c). When exposed PAA, the proportion of dead cells in biofilm samples collected from pilot-scale RAS increased with increasing PAA concentrations, ranging between 17.8 ± 0.6 and 54.0 ± 3.8 %, and a significant rise of dead cells proportion was observed at 16 mg/L PAA (Fig. 3c). The dead cells proportion in biofilm samples collected from commercial RAS altered slightly, ranging from 19.8 ± 0.6 to 25.8 ± 6.0 %, when exposed to elevated PAA concentrations (Fig. 3c).

## 4 Discussion

PAA inhibited endogenous respiration, ammonia oxidation, nitrite oxidation, and enzymatic H_2_O_2_ decomposition processes for biofilter biofilms from pilot-scale RAS. The IC_50_ values showed that nitrite oxidation was more sensitive to PAA than ammonia oxidation, while the endogenous respiration and enzymatic H_2_O_2_ degradation were less affected. This higher sensitivity of nitrification process to PAA is likely due to the nitrifying microbes being aerobic and present at the top layer of biofilm [44,45]. PAA can make contact with the outer layer of biofilm more easily due to PAA diffusion from the bulk liquid phase to biofilm phase, making nitrifying microbes more prone to PAA inhibition compared to microbes in the deeper layers of biofilm. Our finding on nitrite oxidation being more inhibited by PAA than ammonia oxidation is consistent with previous studies [28,[46], [47], [48]]. NOB are known to be more sensitive to environmental perturbations (e.g., oxygen, temperature, pH, and toxicants) than AOB in both natural and engineered systems [[49], [50], [51], [52]].

Endogenous respiration is a fundamental feature of biofilm metabolism, and its rates have been used to indicate the biofilm metabolism related to maintenance energy requirements or active biomass amounts in biofilm [53,54]. All active microorganisms in biofilm carry out endogenous respiration process. Despite the inhibition of AOB and NOB by PAA, the remaining active microorganisms in biofilm can still continue endogenous respiration process to meet their maintenance energy requirements, as indicated by a mild response of endogenous respiration to PAA. Hang et al. [55] also observed that, in response to Cu^2+^ stress, endogenous respiration was the most resilient process compared to the respiration of autotrophic and heterotrophic bacteria in activated sludge.

The microbial activity in biofilm, measured as the rate of H_2_O_2_ decomposition [41,56], was quantified by the rate constant *k*_*or*_. The *k*_*or*_ includes the endogenous oxygen consumption and the oxygen formation caused by enzymatic H_2_O_2_ decomposition [38], depending on both the metabolic activity of the individual microbial cell and the amount of cells in the biofilm. Catalase has been reported to maintain full activity within a completely dead cell in biofilm because it does not require ATP or any regenerated cofactors [57]. When endogenous respiration activity in biofilm was inhibited by PAA, indicating the reduction of active microorganisms and increase of dead cells in biofilm, the oxygen production processes could still continue by catalase-driven H_2_O_2_ decomposition in both live and dead cells in biofilm. This could partly explain why the enzymatic H_2_O_2_ decomposition process in biofilm was the least affected by PAA. Altogether, the observed “hierarchical” responses of biofilm metabolic processes to acute PAA exposure can be attributed to the heterogeneity of biofilm, where different microbial groups occupy distinct ecological niches within the biofilm.

We found that a PAA concentration of 1.27 mg/L led to 50 % inhibition (IC_50_) of nitrite oxidation activity and 1.59 mg/L to 50 % inhibition of ammonia oxidation activity. Both values are 21 % and 31 % lower than previously determined IC_50_ for these processes [46]. This could be due to the difference in applied PAA product composition (i.e., PAA/H_2_O_2_ ratio, 15.7 %/25.8 % vs. 32 %/6 %), the state of tested microbiota (biofilm vs. nitrifying culture), and the methods to evaluate nitrification activity (respirometry vs. batch nitrification assays).

PAA had less effect on the biofilm from the commercial RAS biofilter, compared to the inhibition observed in pilot-scale RAS biofilm. Rates of endogenous respiration and enzymatic H_2_O_2_ decomposition were not suppressed until a concentration of 16 mg/L PAA, at which a significant inhibition of nitrite oxidation and ammonia oxidation occurred. This indicates that biofilter biofilms from commercial RAS have a higher resistance to PAA. One potential explanation of this higher resistance to PAA can be an adaptation from repeated use of PAA in the disinfection practice of the commercial RAS. It is known that repeated exposure to sub-lethal concentrations of disinfectants or antimicrobials can select for a more resistant microbial population in biofilm [[58], [59], [60]]. The possible mechanisms of increased biofilm resistance to disinfectants or antimicrobials compared to suspended bacteria, is due to protection and diffusion limitations of disinfectants or antimicrobials into the biofilm, and could also include phenotypic adaptations of biofilm [61,62]. The unexpected high and variable microbial activity measured at 2 mg/L PAA (Fig. 2d) may support this explanation. However, we cannot rule out the difference in environmental conditions (e.g., light exposure, temperature, DO, and pH) between the two facilities that may have shaped the microbial community in biofilm and lead to different response patterns to PAA.

Liu et al. [48] found that continuous PAA exposure promoted biofilm growth as compared to unexposed systems, however, it was not quantified or tested on bioelements. The biofilm on the bioelements from the commercial RAS was observed to be more rigid compared to the bioelements from the pilot-scale RAS (*data not shown*). Previously, it has been shown that long-term chlorine-based disinfection could cause a more rigid biofilm [63], and non-rigid biofilm could be more easily removed during chlorine disinfection [64]. Therefore, the difference of the mechanical structure of biofilm in two facilities also likely contributed to their different response patterns to PAA.

In addition, both endogenous respiration activity and H_2_O_2_ related microbial activity of unexposed biofilm from commercial RAS was lower than that from pilot-scale RAS. This can be due to the number of active microorganisms in the biofilm from the commercial RAS being lower than that from pilot-scale RAS, as indicated by flow cytometry data. Flow cytometry provided novel information on biofilm viability, which potentially allows to evaluate PAA disinfection efficiency on biofilters. Although concurrent, unexposed controls were included, the exact number of cells detached during the 30-min neutralization step could not be determined. Future studies could quantify detached cells during the neutralization step, such as collecting and analyzing the neutralization solution for viable cell counts. Despite this limitation, the inclusion of triplicate biological replicates ensures the reliability and reproducibility of the observed trends measured by flow cytometry. The flow cytometry results supported the above findings that biofilm samples from commercial RAS had a higher resistance to PAA compared to pilot-scale RAS. Interestingly, flow cytometry results indicate the highest PAA concentration of 16 mg/L only caused around 50 % mortality in biofilm samples from pilot-scale RAS, while a concentration of 2.57 mg/L PAA was already enough to cause 50 % inhibition of endogenous respiration activity of biofilm measured with respirometry method. The different IC_50_ values for biofilm activity and viability indicates that PAA exposure caused a small increase in the proportion of dead cells but a large inhibition of metabolic activity of biofilm, suggesting some microbial cells were viable but not capable to multiply under PAA exposure. This interpretation is supported by a previous study where biofilm cells were found to exhibit significant physiological heterogeneity, with some cells showing reduced metabolic activity while remaining alive in response to antimicrobial exposure [65]. The effect of the two distinct rearing conditions on the microbial composition on the biofilm was not tested but could be one of the reasons why the naive, unexposed pilot-scale RAS biofilm was more susceptible for PAA, compared to the commercial RAS, where the microbial community was adapted to repeated PAA treatment causing ecological shifts in community composition and/or function. Indeed, PAA treatment has been shown to result in major microbial community shift [66], altering microbial composition and structure of biofilms [29,[67], [68], [69]]. Future studies should combine the activity measurements and microbiome analysis to verify whether repeated PAA treatment leads to a less PAA-sensitive microbiome in biofilter biofilms.

The findings of this study provide new information about PAA disinfection in RAS. The use of respirometry and flow cytometry made it possible to quantify biofilm processes and directly compare the effects of PAA exposure. As a result, the inhibition of metabolic processes within biofilter biofilm and disinfection efficiency can be evaluated during PAA disinfection. As for biofilter disinfection between fish batches (terminal disinfection), further increased PAA concentration and exposure time may be required to achieve the desired disinfection.

While respirometry and flow cytometry methods provide valuable insights into the response of aquaculture biofilms to PAA treatment, they do not provide information on the underlying molecular and physiological mechanisms [70]. Specific molecular regulatory mechanisms contributing to the increased resistance of biofilm to PAA, and the effects of PAA on the specific metabolic pathways in biofilms, require advanced methods such as metagenomics, transcriptomics, or proteomics.

## 5 Conclusions

This study quantified the effects of acute PAA exposure on RAS biofilter biofilms. PAA caused a clear dose-dependent inhibition of four selected biofilm processes and on viable cells numbers in biofilm samples from a pilot-scale RAS. In contrast, biofilter biofilms from a commercial RAS were less sensitive to PAA exposure and did not reveal a dose-dependent response to the same PAA exposure range. The results showed that the inhibitory effects of PAA on biofilm were achieved by inhibiting metabolic activities and inactivating viable cells. Further research is required to determine whether previous and regular exposure to PAA, as was the case in the commercial RAS, may also contribute to increasing biofilm resistance to PAA disinfection in pilot-scale RAS. Finally, similar studies with other disinfectants are needed towards improving RAS disinfection strategies with or without the presence of fish.

## CRediT authorship contribution statement

**Wanhe Qi:** Writing – original draft, Visualization, Methodology, Investigation, Formal analysis, Data curation, Conceptualization. **Sanni L. Aalto:** Writing – review & editing, Resources, Methodology. **Peter Vilhelm Skov:** Writing – review & editing, Supervision, Resources, Methodology. **Kim João de Jesus Gregersen:** Writing – review & editing, Supervision. **Lars-Flemming Pedersen:** Writing – review & editing, Supervision, Resources, Project administration, Funding acquisition, Conceptualization.

## Declaration of competing interest

The authors declare that they have no known competing financial interests or personal relationships that could have appeared to influence the work reported in this paper.

## Data Availability

Data will be made available on request.

## References

[1] Flemming H.-C., Wingender J. (2010). The biofilm matrix. Nat Rev Microbiol.

[2] Highmore C.J., Melaugh G., Morris R.J., Parker J., Direito S.O.L., Romero M., Soukarieh F., Robertson S.N., Bamford N.C. (2022). Translational challenges and opportunities in biofilm science: a BRIEF for the future. npj Biofilms Microbiomes.

[3] Lewandowski Z., Beyenal H. (2013).

[4] Martins C.I.M., Eding E.H., Verdegem M.C.J., Heinsbroek L.T.N., Schneider O., Blancheton J.P., d'Orbcastel E.R., Verreth J.A.J. (2010). New developments in recirculating aquaculture systems in Europe: a perspective on environmental sustainability. Aquac Eng.

[5] Moschos S., Kormas K.Ar, Karayanni H. (2022). Prokaryotic diversity in marine and freshwater recirculating aquaculture systems. Rev Aquacult.

[6] Ruiz P., Vidal J.M., Sepúlveda D., Torres C., Villouta G., Carrasco C., Aguilera F., Ruiz-Tagle N., Urrutia H. (2020). Overview and future perspectives of nitrifying bacteria on biofilters for recirculating aquaculture systems. Rev Aquacult.

[7] Rurangwa E., Verdegem M.C.J. (2015). Microorganisms in recirculating aquaculture systems and their management. Rev Aquacult.

[8] Schreier H.J., Mirzoyan N., Saito K. (2010). Microbial diversity of biological filters in recirculating aquaculture systems. Curr Opin Biotechnol.

[9] Qi W. (2024).

[10] Cai W., De La Fuente L., A.C R. (2013). Biofilm formation by the fish pathogen *flavobacterium columnare*: development and parameters affecting surface attachment. Appl Environ Microbiol.

[11] King R.K., Flick G.J., Pierson D., Smith S.A., Boardman G.D., Coale C.W. (2004). Identification of bacterial pathogens in biofilms of recirculating aquaculture systems. J Aquat Food Prod Technol.

[12] Schoina E., Doulgeraki A.I., Miliou H., Nychas G.-J.E. (2022). Dynamics of water and biofilm bacterial community composition in a Mediterranean recirculation aquaculture system. Aquac.

[13] Vidal J.M., Miranda C.D., De la Fuente M., Alarcón M., Aroca G., Sossa K., Ruiz P., Urrutia H. (2020). Formation of biofilms of the salmon pathogen *Flavobacterium psychrophilum* in differents surfaces using the CDC biofilm reactor. Aquaculture.

[14] Holan A.B., Good C., Powell M.D. (2020).

[15] King R.K., Flick G.J., Smith S.A., Pierson M.D., Boardman G.D., Coale C.W. (2008). Response of bacterial biofilms in recirculating aquaculture systems to various sanitizers. J Appl Aquacult.

[16] Pedersen L.F., Pedersen P.B. (2012). Hydrogen peroxide application to a commercial recirculating aquaculture system. Aquac Eng.

[17] Slette H.T., Salomonsen C., Størkersen K., Tveit G.M., Misund A., Lona E. (2024). Biosafety in Norwegian aquaculture—risks and measures in RAS facilities and well-boats. Rev Aquacult.

[18] Lazado C.C., Good C. (2021). Survey findings of disinfection strategies at selected Norwegian and North American land-based RAS facilities: a comparative insight. Aquaculture.

[19] Qi W., Zhu S., Shitu A., Ye Z., Liu D. (2020). Low concentration peroxymonosulfate and UVA-LED combination for *E. coli* inactivation and wastewater disinfection from recirculating aquaculture systems. J Water Proc Eng.

[20] Liu D., Straus D.L., Pedersen L.F., Good C., Lazado C.C., Meinelt T. (2024). Towards sustainable water disinfection with peracetic acid in aquaculture: a review. Rev Aquacult.

[21] Noble A.C., Summerfelt S.T. (1996). Diseases encountered in rainbow trout cultured in recirculating systems. Annu Rev Fish Dis.

[22] Good C., Redman N., Murray M., Straus D.L., Welch T.J. (2022). Bactericidal activity of peracetic acid to selected fish pathogens in recirculation aquaculture system water. Aquac Res.

[23] Liu D., Freches E., Naas C., Behrens S., Meinelt T. (2023). Antibacterial effects of peracetic acid disinfection assessed by culturability, enzyme activity and flow cytometry. Aquaculture.

[24] Meinelt T., Matzke S., Stüber A., Pietrock M., Wienke A., Aj M. (2009). Toxicity of peracetic acid (PAA) to tomonts of *Ichthyophthirius multifiliis*. Dis Aquat Org.

[25] Meinelt T., TM P., Behrens S., Wienke A., LF P., Liu D. (2015). Growth inhibition of *Aeromonas salmonicida* and *Yersinia ruckeri* by disinfectants containing peracetic acid. Dis Aquat Org.

[26] Pedersen L.F., Meinelt T., Straus D.L. (2013). Peracetic acid degradation in freshwater aquaculture systems and possible practical implications. Aquac Eng.

[27] Pinyol-Gallemí A., Pedersen L.-F., Koski M. (2018). Towards control of unwanted cyst-forming dinoflagellates in aquaculture systems: knockout and recovery of *Pfiesteria* sp. after peracetic acid exposure. Aquac Res.

[28] Pedersen L.F., Pedersen P.B., Nielsen J.L., Nielsen P.H. (2009). Peracetic acid degradation and effects on nitrification in recirculating aquaculture systems. Aquaculture.

[29] Suurnäkki S., Pulkkinen J.T., Lindholm-Lehto P.C., Tiirola M., Aalto S.L. (2020). The effect of peracetic acid on microbial community, water quality, nitrification and rainbow trout (*Oncorhynchus mykiss*) performance in recirculating aquaculture systems. Aquaculture.

[30] Yanong R.P.E. (2012).

[31] Kinyage J.P.H., Pedersen L.-F. (2016). Impact of temperature on ammonium and nitrite removal rates in RAS moving bed biofilters. Aquac Eng.

[32] Mnyoro M.S., Arvin E., Munubi R.N., Chenyambuga S.W., Pedersen L.-F. (2021). Effect of water velocity on ammonium and nitrite removal in pilot scale fixed bed biofilters. Aquac Eng.

[33] Davidson J., Summerfelt S., Straus D.L., Schrader K.K., Good C. (2019). Evaluating the effects of prolonged peracetic acid dosing on water quality and rainbow trout *Oncorhynchus mykiss* performance in recirculation aquaculture systems. Aquac Eng.

[34] Aalto S.L., Syropoulou E., de Jesus Gregersen K.J., Tiirola M., Pedersen P.B., Pedersen L.-F. (2022). Microbiome response to foam fractionation and ozonation in RAS. Aquaculture.

[35] Shitu A., Zhu S., Qi W., Tadda M.A., Liu D., Ye Z. (2020). Performance of novel sponge biocarrier in MBBR treating recirculating aquaculture systems wastewater: microbial community and kinetic study. J Environ Manag.

[36] Brown M.N., Briones A., Diana J., Raskin L. (2013). Ammonia-oxidizing archaea and nitrite-oxidizing Nitrospiras in the biofilter of a shrimp recirculating aquaculture system. FEMS Microbiol Ecol.

[37] Qi W., Skov P.V., de Jesus Gregersen K.J., Pedersen L.-F. (2022). Estimation of nitrifying and heterotrophic bacterial activity in biofilm formed on RAS biofilter carriers by respirometry. Aquaculture.

[38] Qi W., Skov P.V., de Jesus Gregersen K.J., Pedersen L.-F. (2023). A novel method to estimate biofilm activity based on enzymatic oxygen release from hydrogen peroxide decomposition. Biofilms.

[39] Aalto S.L., Madsen L., Pedersen L.-F. (2023). Peracetic acid mode-of-action on aquaculture microbes evaluated by dual-staining flow cytometry. Aquaculture.

[40] Aalto S.L., Letelier-Gordo C.O., Pedersen L.-F., Pedersen P.B. (2022). Effect of biocarrier material on nitrification performance during start-up in freshwater RAS. Aquac Eng.

[41] Pedersen L.F., Rojas-Tirado P., Arvin E., Pedersen P.B. (2019). Assessment of microbial activity in water based on hydrogen peroxide decomposition rates. Aquac Eng.

[42] Qi W., Skov P.V., de Jesus Gregersen K.J., Mousavi S., Pedersen L.-F., Mota V.C. (2025). Estimating biofilm activity on biofilter elements in recirculating aquaculture systems (RAS) for rearing Atlantic salmon parr (*Salmo salar*) during operation with ozone and peracetic acid. Aquaculture.

[43] Liu G., Wang D., Wang J., Mendoza C. (2011). Effect of ZnO particles on activated sludge: role of particle dissolution. Sci Total Environ.

[44] Mašić A., Eberl H.J. (2014). A modeling and simulation study of the role of suspended microbial populations in nitrification in a biofilm reactor. Bull Math Biol.

[45] Persson F., Sultana R., Suarez C., Hermansson M., Plaza E., Wilén B.-M. (2014). Structure and composition of biofilm communities in a moving bed biofilm reactor for nitritation–anammox at low temperatures. Bioresour Technol.

[46] Chen J., Song T., Long S., Zhu K.J., Pavlostathis S.G. (2022). Effect of peracetic acid solution on a nitrifying culture: kinetics, inhibition, cellular and transcriptional responses. Water Res.

[47] Lepine C., Redman N., Murray M., Lazado C.C., Johansen L.H., Espmark Å.M., Davidson J., Good C. (2023). Assessing peracetic acid application methodology and impacts on fluidized sand biofilter performance. Aquac Res.

[48] Liu D., Straus D.L., Pedersen L.F., Meinelt T. (2017). Pulse versus continuous peracetic acid applications: effects on rainbow trout performance, biofilm formation and water quality. Aquac Eng.

[49] Downing L.S., Nerenberg R. (2008). Effect of oxygen gradients on the activity and microbial community structure of a nitrifying, membrane-aerated biofilm. Biotechnol Bioeng.

[50] Kim Y.M., Lee D.S., Park C., Park D., Park J.M. (2011). Effects of free cyanide on microbial communities and biological carbon and nitrogen removal performance in the industrial activated sludge process. Water Res.

[51] Németh A., Ainsworth J., Ravishankar H., Lens P.N.L., Heffernan B. (2023). Temperature dependence of nitrification in a membrane-aerated biofilm reactor. Front Microbiol.

[52] Park S., Bae W., Chung J., Baek S.-C. (2007). Empirical model of the pH dependence of the maximum specific nitrification rate. Process Biochem.

[53] Katipoglu-Yazan T., Ubay-Cokgor E., Orhon D. (2021). Acute inhibitory impact of sulfamethoxazole on mixed microbial culture: kinetic analysis of substrate utilization biopolymer storage nitrification and endogenous respiration. Biochem Eng J.

[54] Spanjers H., Vanrolleghem P. (1995). Respirometry as a tool for rapid characterization of wastewater and activated sludge. Water Sci Technol.

[55] Hang Z., Tong P., Zhao P., He Z., Shao L., Jia Y., Wang X.C., Li Z. (2023). Hierarchical stringent response behaviors of activated sludge system to stressed conditions. Sci Total Environ.

[56] Arvin E., Pedersen L.F. (2015). Hydrogen peroxide decomposition kinetics in aquaculture water. Aquac Eng.

[57] Stewart P.S., Owkes M. (2023). Simulation of catalase-dependent tolerance of microbial biofilm to hydrogen peroxide with a biofilm computer model. npj Biofilms Microbiomes.

[58] Capita R., Vicente-Velasco M., Rodríguez-Melcón C., García-Fernández C., Carballo J., Alonso-Calleja C. (2019). Effect of low doses of biocides on the antimicrobial resistance and the biofilms of *Cronobacter sakazakii* and *Yersinia enterocolitica*. Sci Rep.

[59] Maillard J.-Y., Pascoe M. (2024). Disinfectants and antiseptics: mechanisms of action and resistance. Nat Rev Microbiol.

[60] Strantzali D., Kostoglou D., Perikleous A., Zestas M., Ornithopoulou S., Dubois-Brissonnet F., Giaouris E. (2021). Comparative assessment of the disinfection effectiveness of thymol and benzalkonium chloride against adapted and non-adapted to thymol biofilm cells of a *Salmonella Typhimurium* epidemic phage type DT193 strain. Food Control.

[61] Bridier A., Briandet R., Thomas V., Dubois-Brissonnet F. (2011). Resistance of bacterial biofilms to disinfectants: a review. Biofouling.

[62] Mah T.-F.C., O'Toole G.A. (2001). Mechanisms of biofilm resistance to antimicrobial agents. Trends Microbiol.

[63] Shen Y., Huang C., Monroy G.L., Janjaroen D., Derlon N., Lin J., Espinosa-Marzal R., Morgenroth E., Boppart S.A., Ashbolt N.J., Liu W.-T., Nguyen T.H. (2016). Response of simulated drinking water biofilm mechanical and structural properties to long-term disinfectant exposure. Environ Sci Technol.

[64] Guo L., Ye C., Yu X., Horn H. (2023). Induction of bacteria in biofilm into a VBNC state by chlorine and monitoring of biofilm structure changes by means of OCT. Sci Total Environ.

[65] Stewart P.S., Franklin M.J. (2008). Physiological heterogeneity in biofilms. Nat Rev Microbiol.

[66] Chen J., Liu X., Pavlostathis S.G. (2021). Long-term evaluation of the effect of peracetic acid on a mixed aerobic culture: organic matter degradation, nitrification, and microbial community structure. Water Res.

[67] Chino T., Nukui Y., Morishita Y., Moriya K. (2017). Morphological bactericidal fast-acting effects of peracetic acid, a high-level disinfectant, against *Staphylococcus aureus* and *Pseudomonas aeruginosa* biofilms in tubing. Antimicrob Resist Infect Control.

[68] Teitge F., Peppler C., Steinhagen D., Jung‐Schroers V. (2020). Effect of disinfection with peracetic acid on the microbial community of a seawater aquaculture recirculation system for Pacific white shrimp (*Litopenaeus vannamei*). J Fish Dis.

[69] Zhang C., Brown P.J.B., Miles R.J., White T.A., Grant D.G., Stalla D., Hu Z. (2019). Inhibition of regrowth of planktonic and biofilm bacteria after peracetic acid disinfection. Water Res.

[70] Wessels S., Ingmer H. (2013). Modes of action of three disinfectant active substances: a review. Regul Toxicol Pharmacol.

